# A Phase 1 dose-ranging study examining the effects of a superabsorbent polymer (CLP) on fluid, sodium and potassium excretion in healthy subjects

**DOI:** 10.1186/2050-6511-15-2

**Published:** 2014-01-25

**Authors:** Lee W Henderson, Howard C Dittrich, Alan Strickland, Thomas M Blok, Richard Newman, Thomas Oliphant, Detlef Albrecht

**Affiliations:** 1 Sorbent Therapeutics Inc, 710 Lakeway Drive, Suite 290, Sunnyvale, CA 94085, USA; 2 Alan Strickland Consulting, 101 Waterlily, Lake Jackson, TX 77566, USA; 3 Jasper Clinic’s Clinical Research Unit, 526 Jasper Street, Kalamazoo, MI 49007, USA; 4 RnD Services, LLC, 635 Bent Creek Ridge, Deerfield, IL 60015, USA; 5 Innovative Analytics, 161 East Michigan Ave, Kalamazoo, MI 49007, USA

**Keywords:** Superabsorbent polymer, Dose ranging, Pharmacodynamics, Gastrointestinal fluid removal, Gastrointestinal sodium removal

## Abstract

**Background:**

CLP is an orally administered, non-absorbed, superabsorbent polymer being developed to increase fecal excretion of sodium, potassium and water in patients with heart failure and end-stage renal disease. This study was conducted to evaluate the safety of CLP, and to explore dose-related effects on fecal weight, fecal and urine sodium and potassium excretion, and serum electrolyte concentrations.

**Methods:**

This Phase 1, open-label, dose-escalation study included 25 healthy volunteers, who were administered CLP orally immediately prior to four daily meals for 9 days at doses of 7.5, 15.0, and 25.0 g/day (n = 5/group). An additional dose group received 15.0 g/day CLP under fasting conditions, and an untreated cohort (n = 5) served as control. Twenty-four-hour fecal and urinary output was collected daily. Samples were weighed, and sodium, potassium, and other ion content in stool and urine were measured for each treatment group. Effects on serum cation concentrations, other standard laboratory values, and adverse events were also determined.

**Results:**

At doses below 25.0 g/day, CLP was well tolerated, with a low frequency of self-limiting gastrointestinal adverse events. CLP increased fecal weight and fecal sodium and potassium content in a dose-related manner. Concomitant dose-related decreases in urinary sodium and potassium were observed. All serum ion concentrations remained within normal limits.

**Conclusions:**

In this study, oral CLP removed water, sodium and potassium from the body via the gastrointestinal tract in a dose related fashion. CLP could become useful for patients with fluid overload and compromised kidney function in conditions such as congestive heart failure, salt sensitive hypertension, chronic kidney disease and end stage renal disease.

**Trial registration:**

NCT01944007

## Background

Fluid overload and sodium retention are central components in the pathophysiology of heart failure, with up to 90% of hospitalizations in heart failure caused by symptoms and signs of fluid overload [1,2]. There is also growing evidence that hypervolemia *per se* is independently associated with mortality [3,4]. Concomitant renal dysfunction (chronic kidney disease; CKD) has a strong association with poor outcomes in heart failure patients [5,6]. Diuretics are the cornerstone of acute and chronic heart failure therapy, but concerns have been raised about the safety and therapeutic efficacy of high dose diuretic therapy [2,7-9], diuretic resistance in the presence of declining renal function and undesired serum electrolyte effects [10,11]. The use of cationic exchange resins for removal of excess sodium and water via the gastrointestinal tract from edematous patients was under considerable study in the late 1940s and early 1950s [12]. These products failed to achieve a significant therapeutic presence, in large measure due to the high dosage required (up to 150 g/day), problems in taking the large amounts of unpalatable resins, significant abdominal side effects and the advent of loop diuretics. Recently, interest in the use of the gastrointestinal tract to remove fluid and/or ions using polymers of enhanced efficacy and improved tolerability has been renewed [13,14].

New treatment options for fluid and ion management are a significant medical need [2]. CLP (Cross Linked Polyelectrolyte) is a novel, non-absorbed, superabsorbent polymer that, given orally, absorbs water, sodium and potassium in the gastrointestinal tract with eventual elimination in the feces. The clinical effects of CLP in patients with congestive heart failure and impaired kidney function were recently reported in a Phase 2, double-blind, placebo-controlled study. Patients administered 15 g/day CLP lost significantly more weight, had a higher proportion of improvement in New York Heart Association (NYHA) class and showed a trend for improvement in both the six minute walk test and the Kansas City Cardiomyopathy Questionnaire compared to patients receiving placebo [13]. This exploratory Phase 1 study was done before the Phase 2 study and the purpose was to evaluate the safety of CLP, and to explore the dose-related effects on fecal weight, and urine and fecal sodium and potassium excretion.

## Methods

The study was conducted in accordance with International Conference on Harmonisation Good Clinical Practice guidelines and other applicable regulatory requirements and laws. The study protocol was reviewed and approved by the IntegReview Ethical Review Board, Austin, Texas. All subjects provided written informed consent prior to study participation. Trial registration number: NCT01944007.

Healthy volunteers at least 18 years of age considered to be in good general health (no renal, cardiac, hepatic or other major organ dysfunction) and with no prior history of major gastrointestinal surgery, conditions affecting motility, or dyspepsia requiring treatment within the previous 6 months were recruited by the Clinical Pharmacology Research Unit at the Jasper Clinic, Kalamazoo, Michigan.

This Phase 1, open-label, dose-escalation study recruited 25 healthy volunteers who were randomized into five groups (n = 5/group): a) Control (untreated), b) 7.5 g CLP/day, c) 15.0 g CLP/day, d) 15.0 g CLP/day (fasted) and e) 25.0 g CLP/day. CLP was administered orally in size 00 hydroxypropylmethylcellulose capsules immediately prior to meals (breakfast, lunch, dinner, and snack) in 4 doses divided equally for 9 days, except in the 15.0 g/day fasted group who received the medication 1 hour prior to meals to evaluate the effects of CLP on an empty stomach. With the exception of administration of study medication, untreated control subjects were exposed to the same conditions and procedures as CLP subjects. All subjects were given identical, standardized meals that were controlled for the amount of calories, level of sodium (~5000 mg per day ±100 mg), fiber content (10-15 g per day), fat content, and approximate recommended Dietary Reference Intakes. The study subjects were requested to consume all of their meals. Clinic staff monitored and recorded complete ingestion of the meals served during the study. All fluid intake was recorded.

Subjects were admitted to the research unit on Day -1 (baseline); CLP was administered on Days 1 through 9, and subjects were discharged on Day 10.

Fecal and urine samples for each 24-hour period were collected daily during the treatment period and were weighed and analyzed for sodium, potassium, magnesium, calcium, and phosphorus. Daily samples were pooled for analysis. Analyses of ion content in feces were performed by inductively coupled plasma-optical emission spectroscopy (ICP-OES) at Galbraith Labs Inc. (Knoxville, Tennessee). Urine samples and safety laboratories were analyzed at Bronson Methodist Hospital Laboratory (Kalamazoo, Michigan), using standard clinical automated chemistry techniques.

Safety assessments included daily monitoring of adverse events, vital signs and serum chemistries. Hematology, urinalysis, and 12-lead electrocardiogram (ECG) were measured at baseline and prior to discharge. All blood samples were drawn in the fasting stage before breakfast.

### Endpoints

Endpoints to evaluate the pharmacologic activity of CLP included stool weight, fecal content of sodium, potassium, calcium, magnesium and phosphorus, and urine content of sodium, potassium, calcium, magnesium and phosphorus. Safety and tolerability evaluations were based on frequency and severity of adverse events, clinical safety labs and vital signs.

### Statistical analysis

**Figure 1:**
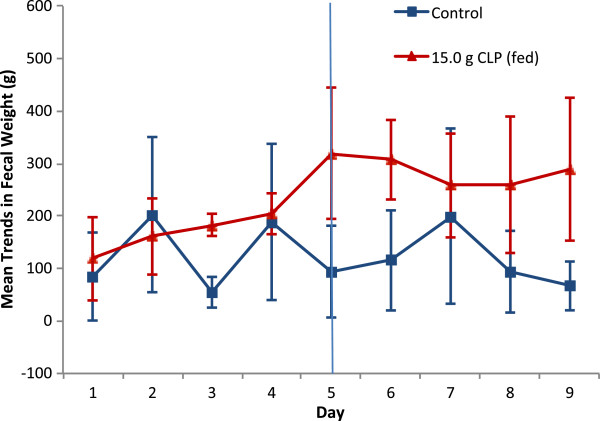
**Daily fecal weight following 15.0 g/day CLP for 9 days under fed conditions.** Values are means ± 95% confidence intervals. Blue line indicates start of steady state.

Fecal ion content, fecal weight, urine ion content and serum ion concentration data were summarized for each CLP dose group and the control group using descriptive statistics for an empirically determined steady state period (Days 5-9). The steady state period is illustrated for stool weight for the 15 g/day fed group in Figure 1. Due to the exploratory nature of the study and the small sample size, no formal confirmatory statistical analysis was performed.

For these descriptive statistical analyses, data collected during the steady-state period were analyzed as the daily average of values measured on Days 5-9. Least squares mean profiles and empirical standard errors for each CLP dose group versus control were estimated via generalized estimating equations in accordance with a 2-group repeated measures analysis of variance (RM-ANOVA) assuming compound symmetry. The least squares means and corresponding 95% confidence intervals from the RM-ANOVA were then used to depict expected cumulative (Days 5-9) mean values for each pairing of CLP versus control. The incidence of treatment-emergent adverse events for each group was determined. Hematology, serum chemistry and quantitative urinalysis analytes were summarized by treatment group using descriptive statistics; qualitative urinalysis analytes were summarized by treatment group. Descriptive statistics were performed on vital signs data for each treatment group. ECG data were summarized for each treatment group as normal or abnormal; abnormal findings were categorized further with regard to clinical significance.

## Results

Subjects ranged in age from 22 to 68 years (mean, 38.0 years). Approximately half of the subjects (56%) were male, and dose groups were not balanced with regard to gender: the control group comprised only females, and all but the 15.0 g/day fed group, which included 2 males and 3 females, had a male to female ratio of 4:1. The sample was predominantly Caucasian (84% of subjects).

### Fecal weight

**Figure 2:**
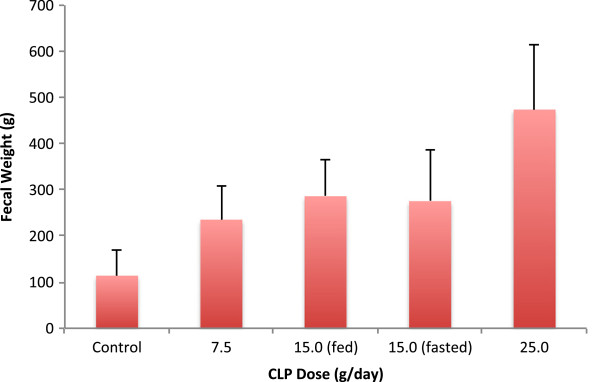
**Mean (standard deviation) fecal weight by treatment group.** Values are daily averages from Days 5-9, the time period reflective of steady state CLP exposure.

Mean fecal weight was higher in all CLP groups compared to the control group during study Days 5-9. Mean fecal weight values increased in a dose-related manner and were similar in the fed and fasted 15.0 g/day CLP groups (Figure 2).

### Fecal ion content

**Figure 3:**
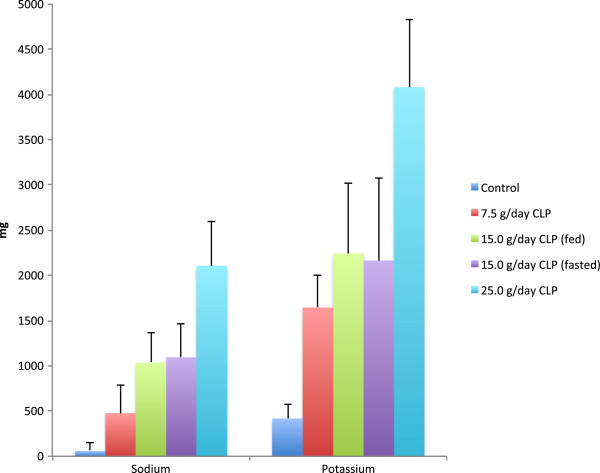
**Mean (standard deviation) fecal content of sodium and potassium following CLP treatment and untreated control.** Values are daily averages from Days 5-9, the time period reflective of steady state CLP exposure.

Mean fecal sodium and potassium content were higher in all CLP groups compared to the control group (Figure 3), and increased in a dose-related manner. Mean fecal magnesium and calcium content were similar across treatment groups and fecal phosphorus was lower than the control group at 15.0 g/day. No differences between fed and fasted state were noted for fecal content of any of the ions.

### Urine ion content

**Figure 4:**
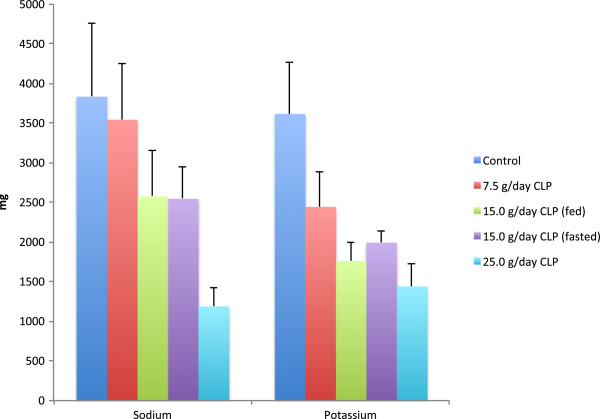
**Mean (standard deviation) urine content of sodium and potassium following CLP treatment and untreated control.** Values are daily averages from Days 5-9, the time period reflective of steady state CLP exposure.

Mean urine sodium and potassium were lower in all CLP groups than the control group, with values in CLP groups showing an inverse relationship to dose (Figure 4). Treatment groups were similar with respect to urine calcium and magnesium, whereas there was an increase in urine phosphorus content at higher doses. There were no noteworthy differences in mean urine cation content values under fed versus fasting conditions.

### Serum ion concentrations

There were no clinically meaningful changes in mean serum ion concentrations during the study. A dose dependent drop in serum potassium resulted in a borderline low serum potassium level in the 25.0 g/day CLP group (Table 1).

**Table 1:** Mean serum ion concentrations during study days 5-9 by treatment group

	**CLP Dose (g/day)**				**Control (n = 5)**
	**7.5 (n = 5)**	**15.0 (Fed) (n = 4)**	**15.0 (Fasted) (n = 5)**	**25.0 (n = 5)**	
Sodium (mmol/L)	141.1 (1.4)	138.5 (1.8)	141.0 (2.6)	137.4 (1.6)	139.4 (1.6)
Potassium (mmol/L)	4.6 (0.3)	4.3 (0.3)	4.3 (0.4)	3.7 (0.2)	4.8 (0.5)
Magnesium (mmoL/L)	0.8 (0.0)	0.8 (0.0)	0.8 (0.0)	0.7 (0.0)	0.9 (0.0)
Calcium (mmol/L)	2.4 (0.0)	2.3 (0.1)	2.4 (0.1)	2.3 (0.1)	2.4 (0.1)
Phosphorus (mmol/L)	1.5 (0.2)	1.4 (0.1)	1.3 (0.1)	1.2 (0.1)	1.6 (0.2)

Values are the mean (standard deviation) of daily averages from Days 5-9.

### Safety

#### Adverse events

No serious adverse events were reported. At doses below 25.0 g/day, CLP was well tolerated. Two subjects in the 25.0 g/day group discontinued CLP dosing on Day 4 due to gastrointestinal adverse events: one due to repeated episodes of moderately severe vomiting, and the other due to abdominal distension, nausea and abdominal pain. Events for both subjects resolved completely without medical treatment prior to discharge on Day 9, five days after termination of dosing. The most frequently reported adverse events were gastrointestinal in nature and appeared to be dose related. However, gastrointestinal adverse events were also reported for 4 of 5 subjects in the control group. The most common gastrointestinal event was nausea. Diarrhea was reported for 1 subject each in the 25.0 g/day CLP and control groups, and no constipation was reported for any of the subjects on CLP, but it was reported for 2 subjects in the control group. All adverse events were mild to moderate in severity, tended to be transient, and resolved without pharmacologic intervention.

### Hematology, clinical chemistries, and other safety assessments

**Figure 5:**
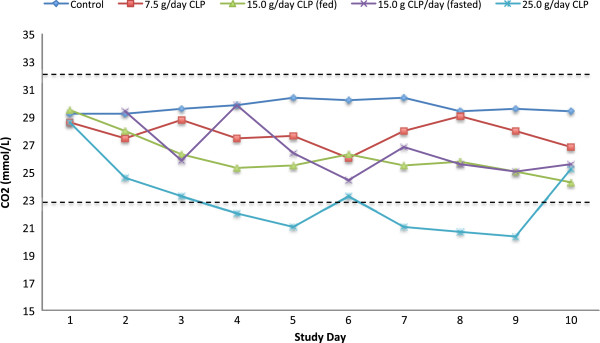
**Mean serum carbon dioxide (CO**_**2**_**) concentration for each treatment group by study day.** Dashed lines represent the normal range (23-32 mmol/L).

No clinically significant changes in mean hematology values were observed in any treatment group. With the exception of the serum carbon dioxide concentration (CO_2_), there were no noteworthy changes in mean clinical chemistry values. As shown in Figure 5, serum CO_2_ decreased as the dose of the acidic CLP polymer increased, and in the 25.0 g/day CLP group, fell below the lower limit of normal on Day 4, and remained decreased until the end of dosing on Day 9. No clinically significant changes in urinalysis were observed, and there were no noteworthy changes in vital signs or ECGs.

## Discussion

This Phase 1 study was conducted to evaluate the tolerability and effects of three different doses of CLP, a novel, non-absorbed superabsorbent polymer, on fecal weight and fecal and urine ion content. No serious adverse events occurred in the 25 subjects studied. At CLP doses below 25.0 g/day, there was a low incidence of self-resolving, mild adverse events. The 25.0 g/day dose immediately prior to meals caused more gastrointestinal adverse events, which resulted in 2 subjects discontinuing the study medication. There was no clinically meaningful difference between groups who received 15.0 g/day CLP under fasting and fed states on any endpoint. Fecal weight, sodium and potassium in the stool were increased relative to control in a dose-related fashion, with a 15.0 g dose removing approximately 3.0 mEq of sodium and 3.4 mEq of potassium per gram of polymer. Serum potassium and serum sodium concentrations were within the normal range for all subjects, with the 25.0 g/day CLP group showing a borderline low potassium level. Serum CO_2_ decreased as the dose of CLP increased; however, only for the 25.0 g/day CLP group did the serum CO_2_ fall below the normal range (23-32 mmol/L).

While there were small changes in fecal phosphorus content, this was considered clinically inconsequential. The reduction in urine sodium and potassium is considered to reflect a normal homeostatic compensation of the kidney for the loss of these cations in the stool [15].

Previous studies with cation exchange resins in the treatment of patients with heart failure and fluid overload demonstrated removal of sodium and potassium via the gastrointestinal route [12]. However, these products failed to achieve a significant therapeutic presence for fluid management, due to the high doses required (up to 150 g/day), problems in taking the large amounts of unpalatable resins, significant abdominal side effects and the advent of loop diuretics. The much higher cation binding capacity of CLP may offer the opportunity to remove up to 1.0 g of sodium with a lower dose of drug, improving efficiency and gastrointestinal tolerability. Moreover, the superabsorbent water binding capacity of CLP allows for direct fluid removal via the fecal route without causing diarrhea.

Removal of sodium and fluid in the feces may prove beneficial in patients with kidney failure and fluid overload, as well as in those with heart failure and CKD where the effectiveness of diuretics is limited and diuretic resistance is an increasing problem [2,7-9]. High dietary sodium intake has been implicated in target organ damage in kidney and heart cells via increases in blood pressure and oxidative stress, reduction of arterial elasticity and fibrotic cell remodeling [16]. Dietary sodium restriction is recommended in the management of hypertension, heart failure and CKD [16-18]. Studies have shown the beneficial effects of dietary sodium reduction to lower blood pressure and reduce deaths from heart attack and stroke [19,20]. In patients with salt sensitive treatment resistant hypertension, systolic blood pressure reductions of over 20 mmHg have been reported after dietary sodium restriction [21]. Sodium reduction also lowered proteinuria in chronic kidney disease patients [22]. However, dietary sodium restriction is difficult to achieve and a 15.0 g/day dose of CLP could remove a clinically meaningful amount of sodium from the systemic circulation [23].

The fecal removal of up to 2.0 g potassium from the body may have desirable benefits for patients at risk for hyperkalemia such as CKD or heart failure patients, especially patients with a glomerular filtration rate of ≤ 60 mL/min who have a higher risk for the occurrence of hyperkalemia [24]. Kayexelate (polystyrene sulfonate), the only approved potassium removing agent, has significant tolerability and safety concerns which would render it inadequate to treat hyperkalemia on a chronic use basis [25,26]. Recently, a study with RLY5016 has demonstrated that hyperkalemia may be prevented with a potassium binding oral polymer [14]. However, in a previously reported Phase 2, double-blind, placebo-controlled study in heart failure patients, no differences were observed between CLP and placebo in serum potassium concentrations. This may have been the result of the concomitant administration of an aldosterone antagonist, which alters serum potassium balance [13].

The main weakness of this study is that the small number of study subjects in each group does not permit formal statistical analysis of differences between groups. A strength of the study is the rigor with which subjects were selected and monitored as inpatients in a metabolic study unit with strict control of total dietary caloric, ion and fluid intake and collection of urine and fecal samples for the duration of the study.

## Conclusions

In this study of healthy volunteers, CLP increased fecal weight and fecal sodium and potassium content in a dose-related manner, and doses below 25.0 g/day were well tolerated, with infrequent, self-limiting gastrointestinal adverse events. CLP may represent a new therapeutic tool for the removal of fluid, sodium and potassium via the gastrointestinal tract in patients with fluid and sodium overload in conditions such as congestive heart failure, salt sensitive hypertension, CKD and end stage renal disease.

## Competing interests

This study was sponsored by Sorbent Therapeutics Inc., Sunnyvale, CA, USA. All authors have completed the Unified Competing Interest form at http://www.icmje.org/coi_disclosure.pdf (http://www.icmje.org/coi_disclosure.pdf) (available on request from the corresponding author) and declare the following: LWH, HCD and DA are employees of Sorbent Therapeutics Inc., Sunnyvale, CA, USA. DA is a stockholder of Relypsa Inc. AS and RN are paid expert advisors engaged by Sorbent Therapeutics, Inc. to support the design, conduct and analysis of this study. TMB is an employee of the Jasper Clinic’s Clinical Research Unit, Kalamazoo, MI, which was engaged by Sorbent Therapeutics, Inc. to conduct this study. TO is an employee of Innovative Analytics, Kalamazoo, MI, which was engaged by Sorbent Therapeutics, Inc. to conduct the data management and statistical analysis for this study. None of the authors received payments for their contributions to the manuscript.

## Authors’ contributions

All authors contributed significantly to the study design (LWH, TMB, AS, RN) and conduct (TMB, TO), data analysis (TO) and interpretation (LWH, AS, RN, HCD, DA). They were involved in the drafting and revising of the manuscript and gave full approval for the final version to be published.

## Pre-publication history

The pre-publication history for this paper can be accessed here:

http://www.biomedcentral.com/2050-6511/15/2/prepub
